# Giant Thyroid Fibrosarcoma- A Case Report

**DOI:** 10.22038/ijorl.2019.39741.2310

**Published:** 2020-03

**Authors:** Manuel Tucciarone, Carlos Heredia-Llinas, Alejandro Lowy-Benoliel, Rosalia Souviron-Encabo, Tomas Martínez- Guirado, Ricardo González-Orus Álvarez-Morujo

**Affiliations:** 1 *Department of Otorhinolaryngology, Gregorio Marañón Hospital, Madrid, SPAIN. *

**Keywords:** Fibrosarcoma, Head and Neck, Head and Neck Cancer, Neoplasm, Thyroid cancer

## Abstract

**Introduction:**

Thyroid gland fibrosarcomas are extremely rare tumors, and only very few cases have been described in the literature. There are no set recommendations along with follow-ups regarding the treatment of these tumors. Moreover, the prognosis is poor with a very short life expectancy.

**Case Report:**

We present an 81-year-old patient who was suffering from increasing dysphagia and dysphonia related to a painless giant cervical mass, which presented with progressive growth for the preceding months. After a core needle biopsy with a suspicion of a solitary fibrous tumor, total excision of the tumor was successfully performed, and the pathology examination revealed a fibrosarcoma. Following surgery, radiotherapy was decided in the oncological multidisciplinary meeting; however, the patient refused it. There were neither new clinical symptoms nor tumor recurrence after an 18-month follow-up.

**Conclusion:**

Although it is a very rare tumor, primary fibrosarcoma of the thyroid gland should be kept in mind in the differential diagnosis of neck tumors.

## Introduction

Nonepithelial cancers of the thyroid gland are very uncommon representing less than 1% of the total thyroid cancers (1). Primary fibrosarcoma of the thyroid gland has been rarely reported in the literature and generally presented as a painless growing tumor causing breathing and swallowing difficulties due to tumoral compression of the neck structures. Computerized tomography (CT), ultrasound (US), and magnetic resonance imaging (MRI) are useful for diagnosis; however, there are no specific findings for this tumor. The definitive diagnosis needs to be established with histopathological examination. There are no general recommendations and follow-ups regarding therapy. The treatment strategies include surgery with or without chemotherapy and local radiation or palliative care in advanced cases (2). The present study reports a case of an 81-year-old male patient diagnosed with primary thyroid fibrosarcoma; moreover, this study reviews the literature in this regard.

## Case Report

**Fig 1:**
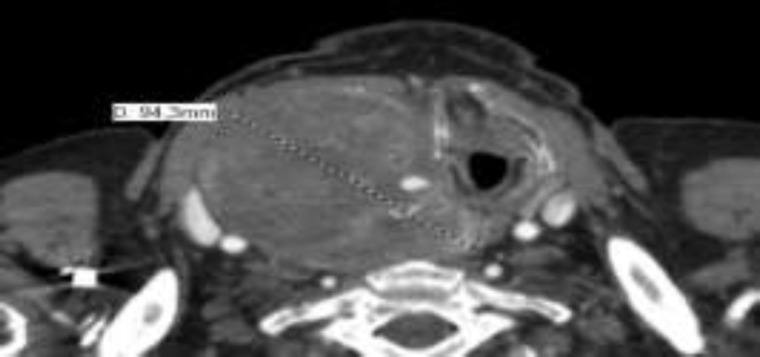
CT scan image showing a giant mass with narrowing and contralateral displacement of larynx and trachea

This study presents a rare case of an 81-year-old male with an unremarkable medical history who was admitted to our department complaining of increasing dysphagia and dysphonia caused by a giant cervical mass which had grown steadily during the previous six months. Clinical examination showed a solid, painless, mobile mass extending to the right and central areas of the neck. Ultrasound examination revealed a heterogeneous hypoechoic mass with irregular edges. The CT scan confirmed a giant, heterogeneous, and hypodense mass (95×72×145mm) extending from the hyoid bone to the retrosternal area with compression, narrowing, and left displacement of larynx and trachea. There was also no evidence of locoregional or metastatic disease (Fig.1). 

Laboratory findings revealed that the patient was euthyroid. Fine needle aspiration biopsy (FNAB) was inconclusive, and a core needle biopsy was performed with a suspicion of a solitary fibrous tumor; therefore, we performed a surgical excision of the tumor. We approached the neck with right cervicotomy, and it was possible to identify and dissect the vascular pedicle of the tumor, which was attached to the right thyroid lobe. The mass was macroscopically removed while preserving the thyroid gland (^Fig’s 2^, 3). 

Postop was uneventful, and the histopathological and immunohistochemical studies established the final diagnosis of fibrosarcoma (G2: moderately differentiated). 

**Fig 2:**
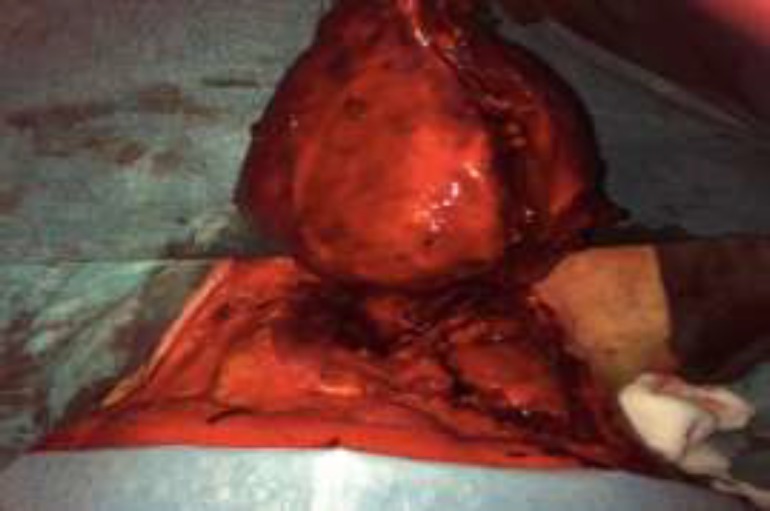
Intraoperative image showing the giant mass with its vascular pedicle attached to the right thyoid lobe

**Fig 3:**
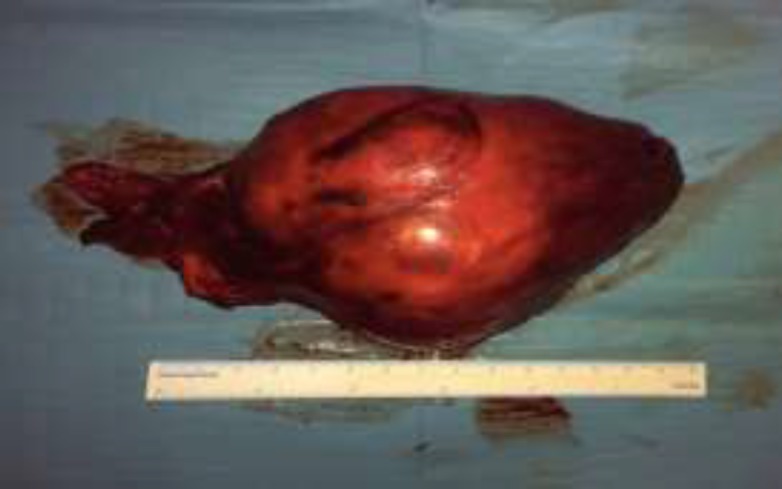
Fibrosarcoma after complete resection

**Fig4:**
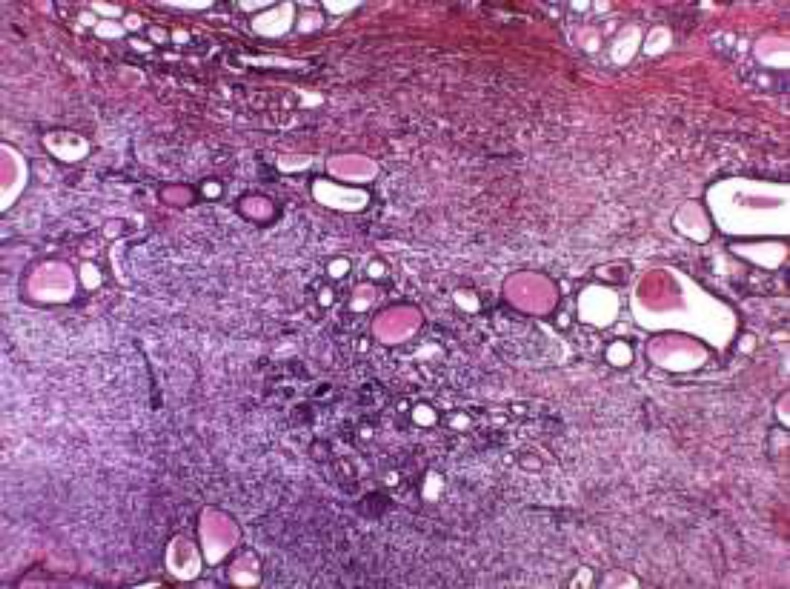
Slice from the histopathological examination with Hematoxylin and eosin staining revealing a proliferation of ovoid spindle atypical cells accompanied with lots of collagen fibers and blood vessels with irregular borders

Histopathological examination with hematoxylin-eosin techniques revealed a proliferation of ovoid spindle atypical cells associated with abundant collagen fibers and blood vessels with irregular edges. Numerous mitotic figures were present and the mean necrosis percentage was inferior to 50% (Fig.4). 

In immunohistochemical staining, the spindle cell areas were focally immunoreactive to CD34, BCL2, and CD99. However, they had no reactivity to CK, desmin, S-100, STAT6, CD31, TTF-1, AE1/AE3, EMA, and HMB-45. In the head and neck, oncological multidisciplinary team complementary treatment with radiotherapy was decided although the patient refused it. After one year follow-up, there were no clinical or radiological signs of recurrence.

## Discussion

Papillary carcinoma is the most frequent thyroid malignancy followed by follicular carcinoma. Less frequent thyroid malignancies of epithelial origin include medullary carcinoma and Hurthle cell carcinoma. Anaplastic thyroid cancer is very rare; however, it is an aggressive variant of thyroid malignancy (3). Nonepithelial cancers, such as fibrosarcoma, are uncommon thyroid malignancies, which represent less than 1% of all thyroid cancers (1). Fibrosarcomas arise from organs containing fibrous tissue most frequently affecting limbs and trunk (4). It is extremely rare for the thyroid gland to be the primary origin of these tumors. Surov et al. showed that fibrosarcomas represented up to 9.2% of all thyroid sarcomas, and they were more frequent in older patients and tended to present a female predominance in the analyzed cases.Clinically, thyroid sarcomas present as painless large goiter with a variable growth rate (in some cases it grows rapidly, whereas it has a slow progressive increase in size among others), which can be accompanied by dyspnea, cough, and dysphagia caused by compression or infiltration of adjacent structures (2). Normal thyroid function, as in the patient we present, has also been described in the literature (5). Fibrosarcomas have a higher rate of lymph node metastases, however, at the same time, an inferior rate of distant metastases or bordering structures infiltration was reported, compared to other variants of primary thyroid sarcomas (2).

Although the US, CT, and MRI can offer useful information regarding size, compression, or infiltration of adjacent structures, they do not lead to specific diagnostic findings (6).

According to the findings obtained from the US, the mass was heterogeneous and hypoechoic in our patient, whereas the CT scan revealed a hypodense nodule in which it was possible to observe displacement and narrowing of the superior aerodigestive tract. In our case, FNAB was inconclusive which made the core needle biopsy the most important preoperative management test although the report suggested a solitary fibrous tumor. The FNAB is generally useful to distinguish epithelial, histiocytic, lymphoid, or mesenchymal origin. 

Nevertheless, a definitive diagnosis can only be reached with histopathological and immunohistochemical analysis (7). Prognosis depends on tumor cell structure, pleomorphism, mitotic activity, and necrosis. Negative reactivity to the European Medicines Agency supports the differential diagnosis from carcinoma and negative staining with HMB-45 plays an important role in the differential diagnosis of malignant melanoma and clear cell carcinoma (7). 

In contrast with epithelial thyroid tumors, there are no general recommendations concerning the treatment and follow-up of sarcomas. Surgery is generally accepted to be the main therapeutical weapon; moreover, chemotherapy and radiotherapy have been used above all in cases of subtotal resections or in situations of infiltration of adjacent structures; however, there are controversies in terms of their benefits. Prognosis is very poor with a mean life expectancy of a few months after surgery (5-8). Even though the complementary treatment has not been performed, our patient did not suffer any recurrence after an 18-month follow-up. 

In conclusion, primary thyroid fibrosarcomas are extremely rare though we must always bear them in mind when confronting anterior neck masses. 

Surgery is the mainstay of treatment in thyroid sarcomas, whereas the use of chemotherapy and radiotherapy remains still unclear. In this case, we show the particularity of a patient in whom only surgery was performed with no recurrence demonstrated after 18 months from the treatment.
